# Neurochemical and Neurotoxic Effects of MDMA (Ecstasy) and Caffeine After Chronic Combined Administration in Mice

**DOI:** 10.1007/s12640-017-9831-9

**Published:** 2017-11-13

**Authors:** Anna Maria Górska, Katarzyna Kamińska, Agnieszka Wawrzczak-Bargieła, Giulia Costa, Micaela Morelli, Ryszard Przewłocki, Grzegorz Kreiner, Krystyna Gołembiowska

**Affiliations:** 1 Department of Pharmacology, Institute of Pharmacology, Polish Academy of Sciences, Smętna 12 Street, 31-343 Kraków, Poland; 2 Department of Molecular Neuropharmacology, Institute of Pharmacology, Polish Academy of Sciences, Kraków, Poland; 3 Department of Biomedical Sciences, Section of Neuropsychopharmacology, University of Cagliari, Cagliari, Italy; 4 Department of Brain Biochemistry, Institute of Pharmacology, Polish Academy of Sciences, Kraków, Poland

**Keywords:** Dopamine, Serotonin, Microdialysis, Oxidative stress, Mice, MDMA, Caffeine

## Abstract

MDMA (3,4-methylenedioxymethamphetamine) is a psychostimulant popular as a recreational drug because of its effect on mood and social interactions. MDMA acts at dopamine (DA) transporter (DAT) and serotonin (5-HT) transporter (SERT) and is known to induce damage of dopamine and serotonin neurons. MDMA is often ingested with caffeine. Caffeine as a non-selective adenosine A1/A2A receptor antagonist affects dopaminergic and serotonergic transmissions. The aim of the present study was to determine the changes in DA and 5-HT release in the mouse striatum induced by MDMA and caffeine after their chronic administration. To find out whether caffeine aggravates MDMA neurotoxicity, the content of DA and 5-HT, density of brain DAT and SERT, and oxidative damage of nuclear DNA were determined. Furthermore, the effect of caffeine on MDMA-induced changes in striatal dynorphin and enkephalin and on behavior was assessed. The DA and 5-HT release was determined with in vivo microdialysis, and the monoamine contents were measured by HPLC with electrochemical detection. DNA damage was assayed with the alkaline comet assay. DAT and SERT densities were determined by immunohistochemistry, while prodynorphin (PDYN) and proenkephalin were determined by quantitative PCR reactions. The behavioral changes were measured by the open-field (OF) test and novel object recognition (NOR) test. Caffeine potentiated MDMA-induced DA release while inhibiting 5-HT release in the mouse striatum. Caffeine also exacerbated the oxidative damage of nuclear DNA induced by MDMA but diminished DAT decrease in the striatum and worsened a decrease in SERT density produced by MDMA in the frontal cortex. Neither the striatal PDYN expression, increased by MDMA, nor exploratory and locomotor activities of mice, decreased by MDMA, were affected by caffeine. The exploration of novel object in the NOR test was diminished by MDMA and caffeine. Our data provide evidence that long-term caffeine administration has a powerful influence on functions of dopaminergic and serotonergic neurons in the mouse brain and on neurotoxic effects evoked by MDMA.

## Introduction

MDMA (3,4-methylenedioxymethamphetamine) known as “ecstasy” is one of the most popular illicit drugs with empathogenic properties. MDMA acting at dopamine (DA) transporter (DAT) and serotonin (5-HT) transporter (SERT) stimulates non-exocytotic release of DA and 5-HT (Baumann et al. 2005; Sulzer et al. 2005). In rodents, MDMA has a preferential affinity for SERT over DAT, so it exerts a more pronounced effect on 5-HT release (Rudnick and Wall 1992). After entering the cell via intracellular transport or diffusion, MDMA alters vesicular monoamine transporter (VMAT-2) and causes an increase in cytoplasmic DA and 5-HT concentrations (Sulzer et al. 2005). This effect is intensified by inhibition of monoamine oxidase type B (MAO-B) located in the outer membrane of the mitochondria of serotonergic neurons (Leonardi and Azmitia 1994). MDMA has been shown to elicit long-lasting neurotoxic effects which vary depending on gender and strain of animals (Brodkin et al. 1993; Colado et al. 2001; Costa et al. 2013; McCann et al. 2005). MDMA and its metabolites are thought to contribute to reactive oxygen species (ROS) formation and to cause selective 5-HT neuron damage in rats (de la Torre and Farre 2004; Sprague and Nichols 2005). Similarly, the damage in serotonergic system was also observed in non-human primates and in the human brain (Green et al. 2003; Ricuarte et al. 1988).

Numerous complex mechanisms have been identified as contributors to the neurotoxic effects of MDMA. Oxidative stress and excitotoxicity represent important mechanisms causing neuronal damage by MDMA (Cadet et al. 2001). MDMA has also been shown to trigger neuroinflammation which seems to be linked with glial activation, in particular microglial activation (Costa et al. 2013, 2014; Frau et al. 2013; Lopez-Rodriguez et al. 2014). Other putative mechanisms of MDMA neurotoxicity include hyperthermia, metabolic toxic products, and apoptosis (Capela et al. 2007; Green et al. 2004).

Toxic and inflammatory effects of MDMA are exacerbated by its co-administration with other psychoactive substances (Khairnar et al. 2010, 2014). Caffeine is commonly consumed with MDMA in energy drinks to reduce drowsiness and fatigue, or it is present in illicit drug preparations, e.g., in ecstasy pills (Peacock et al. 2017). Molecular mechanism of caffeine action in the brain is based on adenosine receptor antagonism. The targets of caffeine actions include G-protein-coupled adenosine A1 and A2A receptors. In a microdialysis study, perfusion with caffeine caused an increase in DA release which was mimicked by the selective A1 receptor antagonist DPCPX, while A1 receptor agonists, but not adenosine A2A agonists, depressed DA levels (Okada et al. 1997). It is also suggested that adenosine A1 receptors present on glutamatergic neurons may be involved in striatal DA release (Borycz et al. 2007).

Caffeine in high doses elevates messenger RNA (mRNA) coding for opioid neuropeptides deriving from prodynorphin (PDYN) and proenkephalin (PENK) (Svenningsson et al. 1997). Medium-sized spiny GABA neurons projecting to the substantia nigra express D1 receptor as well as neuropeptides derived from PDYN (Vincent et al. 1982). On the other hand, medium-sized spiny neurons projecting to the globus pallidus express D2 receptor (Gerfen et al. 1990) as well as PENK-derived peptides (Curan and Watson 1995). DA release increased by amphetamines activates neurons that express D1 receptors and PDYN, but not those that express D2 receptors and PENK (Granado et al. 2014; Johansson et al. 1994). By contrast, caffeine increases the activity of both types of neurons (Johansson et al. 1994).

Caffeine augmented many effects associated with MDMA use. The hyperthermia produced by MDMA in rats was exacerbated by caffeine (McNamara et al. 2006; Vanattou-Saïfoudine et al. 2010a, b). MDMA-induced hyperthermia and tachycardia but not hyperlocomotion were promoted by caffeine in rats. The activation of D1 receptors and A2A receptors and phosphodiesterase (PDE) inhibition were proposed as mechanisms of these synergistic interactions (Vanattou-Saïfoudine et al. 2010a, b, 2012). Other studies showed that administration of caffeine prior to MDMA in mice potentiated the increase in locomotor activity induced by MDMA (Camarasa et al. 2006) and enhanced MDMA-induced DA release in rat striatal slices via blockade of A1 receptors (Vanattou-Saïfoudine et al. 2011). Our earlier results indicated that exacerbation of MDMA effect on DA and 5-HT release by caffeine in mice was mediated via adenosine A1 and A2A receptors (Górska and Gołembiowska 2015). The results of Khairnar et al. (2010) and Frau et al. (2016) demonstrated that neuroinflammatory response induced by MDMA in adult C57BL/6J mice and mice treated in adolescence with MDMA was potentiated by caffeine. The abovementioned data suggest that caffeine increases MDMA-related neurotoxicity. On the other hand, the results of Ruiz-Medina et al. (2013) showed that 22-day pretreatment with a low dose of caffeine completely prevented MDMA-induced neuroinflammation in CD1 mice. This raises the question about a long-term caffeine effect on MDMA neurotoxicity.

The aim of the current investigation was to determine changes in DA and 5-HT release in the mouse striatum induced by MDMA and caffeine. Both drugs were administrated repeatedly in a way mimicking recreational use by young people in dance clubs. To find out whether caffeine facilitates MDMA neurotoxicity, the content of DA and 5-HT, the density of brain DA and 5-HT transporters as markers of nerve terminal damage, and the oxidative damage of nuclear DNA were determined. Since D1 receptors present in medium-sized spiny GABA neurons projecting to the substantia nigra seem to play a role in MDMA neurotoxicity, the effect of caffeine on MDMA-induced changes in PDYN and PENK gene expressions was also examined. In addition, because caffeine could affect behavioral responses, we assessed the effect of chronic caffeine administration on some behavioral parameters associated with MDMA administration.

## Materials and Methods

### Animals

Experiments were performed on adult male (8–10 weeks old) C57BL/6J inbred mice. The animals were housed 5–7 per cage, under a 12-h light/12-h dark cycle, with free access to standard food and tap water. The experiments were conducted in accordance with the European Union guidelines regarding the care and use of laboratory animals (Council Directive 86/609/EEC of November 24, 1986) and were approved by the II Local Bioethics Commission (Kraków, Poland).

### Drugs and Reagents

Caffeine (CAF), was obtained from Sigma-Aldrich (Poznań, Poland) while MDMA was purchased from Toronto Research Chemicals Inc. (Canada). Ketamine hydrochloride and xylazine hydrochloride were supplied by Biowet (Puławy, Poland). Caffeine and MDMA were dissolved in 0.9% NaCl. All injections were done via intraperitoneal (ip) route, and control animals received their respective vehicles. The chemicals used for high-performance liquid chromatography (HPLC) were purchased from Merck (Warsaw, Poland).

### Pattern of Drug Administration

Mice received repeated doses of MDMA (4 × 10 mg/kg every 2 h) for 2 days ip and CAF (2 × 5 mg/kg every 4 h) for 2 days ip. After 5 days free of MDMA treatment, the animals received the next series of “binge” injections. Between binge injections, animals received caffeine (2 × 5 mg/kg) or saline for 5 days. This cycle of treatments was repeated 3 times as shown on the diagram below.

### Brain Microdialysis

Animals were anesthetized with ketamine (7.5 mg/kg) and xylazine (1 mg/kg), and vertical microdialysis probes were implanted into the striatum using the following coordinates: AP + 1.0, L + 1.8, and V − 3.8 (Paxinos and Franklin 2008). On the next day, probe inlets were connected to a syringe pump (BAS, IN, USA) which delivered an aCSF composed of (mM) NaCl 147, KCl 2.7, MgCl_2_ 1.0, and CaCl_2_ 1.2, at pH 7.4 at a flow rate of 1.5 μl/min. After 1 h of the washout period, three basal dialysate samples were collected every 30 min; then, animals were injected with a challenging dose of an appropriate drug as indicated in the figure captions and fraction collection continued for 360 min. At the end of the experiment, the mice were sacrificed and their brains were histologically examined to validate the probe placement.

### Analytical Procedure

DA and 5-HT contents in dialysate fractions were analyzed by HPLC with coulochemical detection. Chromatography was performed using an Ultimate 3000 System (Dionex, USA), coulochemical detector Coulochem III (model 5300, ESA, USA) with 5020 guard cells, 5014B microdialysis cell, and Hypersil Gold-C18 analytical column (3 × 100 mm). The mobile phase was composed of 0.1 M potassium phosphate buffer adjusted to pH 3.6, 0.5 mM EDTA, 16 mg/l 1-octanesulfonic acid sodium salt, and 2% methanol. The flow rate during analysis was set at 0.7 ml/min. The applied potential of a guard cell was + 600 mV, while those of microdialysis cells were *E*_1_ = − 50 mV and *E*_2_ = + 300 mV with a sensitivity set at 50 nA/V. The chromatographic data were processed by Chromeleon v. 6.80 (Dionex, USA) software, run on a personal computer.

### The Tissue Content of DA, 5-HT, and Their Metabolites

Animals were sacrificed by decapitation 3 h after cessation of treatment with drugs. Brains were separated, and several brain regions (striatum, frontal cortex) were dissected in anatomical borders. The tissue levels of DA, 5-HT, 3,4-dihydroxyphenylacetic acid (DOPAC), homovanillic acid (HVA), and 5-hydroxyindoleacetic acid (5-HIAA) were measured using a HPLC with electrochemical detection. Briefly, tissue samples of brain structures were homogenized in ice-cold 0.1 M HClO_4_ and were centrifuged at 10000×*g* for 10 min at 4 °C. The supernatant (3–5 μl) was injected into the HPLC system. The chromatography system consisted of an LC-4C amperometric detector with a cross-flow detector cell (BAS, IN, USA), an Ultimate 3000 pump (Thermo Scientific, USA), and a Hypersil Gold analytical column (3 μm, 100 × 3 mm, Thermo Scientific, USA). The mobile phase consisted of 0.1 M KH_2_PO_4_, 0.5 mM Na_2_EDTA, 80 mg/l sodium 1-octanesulfonate, and a 4% methanol, adjusted to pH 3.7 with an 85% H_3_PO_4_. The flow rate was 1 ml/min. The potential of a 3-mm glassy carbon electrode was set at 0.7 V with sensitivity of 5 nA/V. The temperature of the column was maintained at 30 °C. The Chromax 2007 program (Pol-Lab, Warszawa, Poland) was used for data collection and analysis.

### Comet Assay

#### Preparation of Nuclear Suspension

Animals were killed 60 days after termination of drug treatments. The whole cortex was separated in anatomical borders. Next, the brain tissue was minced with surgical scalpel and homogenized in a manual homogenizer with homogenizing solution containing 0.25% Triton. The homogenate was filtered and centrifuged at 850×*g* for 10 min. The supernatant was discarded while the pellet was resuspended in the same volume of homogenization medium without Triton and centrifuged at 850×*g* for 10 min. The sediment was washed once more in the same way and centrifuged at 600×*g* for 8 min. The pellet was resuspended in 0.8 ml of homogenization solution without Triton, mixed with 4.2 ml of purification medium, and centrifuged at 19,000×*g* for 45 min. The nuclei were obtained as a transparent sediment at the bottom. The pellet was resuspended in 0.5 ml of 2.0 M sucrose and was layered over a sucrose gradient (2.6 and 2.4 M bottom to top). The gradient was allowed to stand for 3 h at 0 °C before use. Nuclear fractionation was obtained by centrifugation at 19,000×*g* for 45 min.

#### Alkaline Comet Assay

The nuclei were added to a tube with 200 μl of PBS (without Ca^++^ and Mg^++^) and mixed gently. The suspension was mixed with LMA agarose and transferred immediately onto Comet slides. The slides were placed at 4 °C in the dark for 10 min. Then, the slides were immersed in pre-chilled lysis solution and left at 4 °C in the dark for 30 min. The buffer was drained, and the slides were immersed in alkaline unwinding solution and were left for 45 min in the dark. Next, electrophoresis was run at 21 V for 30 min. After electrophoresis, the slides were washed first with H_2_O then with 70% ethanol and dried at 45 °C for 10 min. The slides were then covered with dye and allowed to dry completely at room temperature in the dark. On the next day, the slides were examined under a fluorescent microscope. DNA damage was presented as an olive tail moment. Olive tail moment is defined as the product of the tail length and the fraction of total DNA in the tail. Tail moment incorporates a measure of both the smallest detectable size of migrating DNA (reflected in the comet tail length) and the number of damaged pieces (represented by the intensity of DNA in the tail). The value of olive tail moment was calculated according to the following formula: olive tail moment = (tail_mean_ − head_mean_) × tail%DNA/100.

### Prodynorphin and Proenkephalin Levels in the Mouse Striatum

#### Tissue Collection and RNA Isolation

Brains were removed from the skull, and tissue samples including the striatum were collected. Samples were placed in individual tubes with the tissue storage reagent RNAlater (Qiagen Inc., Valencia, CA, USA) and stored at − 70 °C until RNA isolation. Samples were thawed at room temperature and homogenized in 1 ml of Trizol reagent (Invitrogen, Carlsbad, CA, USA). RNA isolation was performed in accordance with the manufacturer’s protocol. The total RNA concentration was measured using a NanoDrop ND-1000 spectrophotometer (NanoDrop Technologies Inc., Montchanin, DE, USA). Reverse transcription (RT) was performed on 1000 ng of total RNA using Omniscript reverse transcriptase (Qiagen Inc.) at 37 °C for 60 min. Reverse transcriptase reactions were performed in the presence of an RNase inhibitor (rRNAsin; Promega, Madison, WI, USA) and oligo (dT)12–18 primer (Invitrogen).

#### Quantitative PCR

The qPCR reactions were performed using assay-on-demand TaqMan probes, hypoxanthine guanine phosphoribosyltransferase (HPRT) (Mm00446968_m1), PDYN (prodynorphin Mm0045753_m1), and PENK (proenkephalin Mm01212875_m1; Applied Biosystems, Carlsbad, CA, USA), and were run on the CFX96 Touch real-time PCR machine (BioRad, Hercules, CA, USA). The expression of the HPRT1 transcript was quantified at a stable level between the experimental groups to control for variations in cDNA amounts. Cycle threshold values were calculated automatically by iCycler IQ 3.0 software with default parameters. Abundance of RNA was calculated as 2 − (threshold cycle).

### DAT and SERT Densities in the Mouse Striatum

#### Immunohistochemistry Studies

##### Tissue Preparation

Coronal brain sections were cut at 50 μm on a vibratome and stored in a cryoprotectant solution at − 20 °C until use. For each mouse, three sections were collected from each of the brain regions analyzed at the following coordinates: from 2.22 to 1.78 mm (frontal cortex) and from 1.34 to 0.74 mm (striatum) relative to bregma, according to the mouse brain atlas of Paxinos and Franklin (2008).

##### Reaction Protocols

Free-floating sections were rinsed in 0.1 M phosphate buffer (PB), blocked in a solution containing 3% normal donkey serum (Jackson ImmunoResearch Europe, Suffolk, UK) and 0.3% Triton X-100 in 0.1 M PB at room temperature for 2 h, and incubated in the same solution with the primary antibody (monoclonal rat anti-DAT antibody diluted 1:1000, Millipore, Temecula, CA, USA; polyclonal rabbit anti-SERT antibody diluted 1:1000, Chemicon, Temecula, CA, USA) for two nights. After completion of incubation with the primary antibody, sections were rinsed three times in 0.1 M PB and incubated with the secondary antibody (AlexaFluor® 594-labeled donkey anti-rabbit diluted 1:400 and AlexaFluor® 488-labeled donkey anti-rat diluted 1:400, Jackson ImmunoResearch Europe, Suffolk, UK) in 0.1 M PB at room temperature for 2 h. After incubation with the secondary antibody, sections were rinsed and immediately mounted onto glass slides coated with gelatin in Mowiol mounting medium.

##### Image Analysis

Images of single wavelengths were obtained with an epifluorescence microscope (Axio Scope A1, Zeiss, Oberkochen, Germany) connected with a digital camera (1.4 MPixels, Infinity 3–1, Lumenera, Nepean, Canada). In each of the three brain sections, two portions from the striatum (dorsolateral and ventromedial) or the prelimbic/infralimbic areas of the medial prefrontal cortex (mPFC), left and right, were acquired using a × 20 objective. For both DAT and SERT immunoreactivities in the striatum and mPFC, the density of immunoreacted fibers was determined quantitatively using the ImageJ software (US National Institutes of Health, USA). Sections were captured in black and white 8-bit monochrome, and the density of fibers was determined in fixed regions using a threshold level that was kept constant across all images. The pixels were converted into square micrometers by employing a suited calibration, in order to represent the area occupied by a specific immunoreaction product in square micrometers. The final values are expressed as a percentage of the respective vehicle group. No significant differences in the density of immunoreacted fibers were seen between the three coronal sections. For each level of the striatum and mPFC, the obtained value was first normalized with respect to the vehicle, then, values from different levels were averaged.

### Behavioral Tests

#### Exploratory and Locomotor Activities in the Open-Field Test

The test was performed using a black PCV box (67 cm × 57 cm × 30 cm, length × width × height) divided into six symmetrical sectors. The arena was dimly illuminated with an indirect light of 18 lx. Each experiment was carried out during the light phase of the light/dark cycle. The mice were selected from separate housing cages. Each mouse was diagonally placed in the middle of the box. The behavior of the animals (line crossing, center square duration, rearing, stretch attend postures) was measured over a 5-min period. The test box was wiped clean between each trial.

#### Novel Object Recognition Test

New object recognition test in mice was carried out using a wooden, black, round “free-field,” placed 80 cm above the floor with a diameter of 100 cm and divided into eight equal sectors with white line. The laboratory room was dark, and only the center of the open field was illuminated with a 25-W bulb placed 75 cm above the platform. On the first day of the experiment (adaptation), mice were placed in the open field for 10 min. On the next day, the animals were placed in the open field for 5 min with two identical objects (white tin, 5 cm wide and 14 cm high or green pyramid 5 cm wide and 14 cm high). The time of object interest was measured for each of the two objects separately. Then, 1 h after the first session, the mice were again placed in a free field for 5 min with two different objects, one from the previous session (old) and the other new (white box and green pyramid). The time of object interest was measured for each of the two objects separately (sniffing, touching, or climbing).

### Data Analysis

All obtained microdialysis data were presented as a percent of the basal level assumed to be 100%. The statistical significance was calculated using a repeated-measure ANOVA or where appropriate a one-way ANOVA, followed by Tukey’s post hoc test. The results were considered statistically significant when *P* < 0.05.

## Results

### The Effect of Repeated “Binge” Administration of MDMA, Caffeine, and Their Combination on the Extracellular Level of DA and 5-HT in the Mouse Striatum

**Fig. 1:**
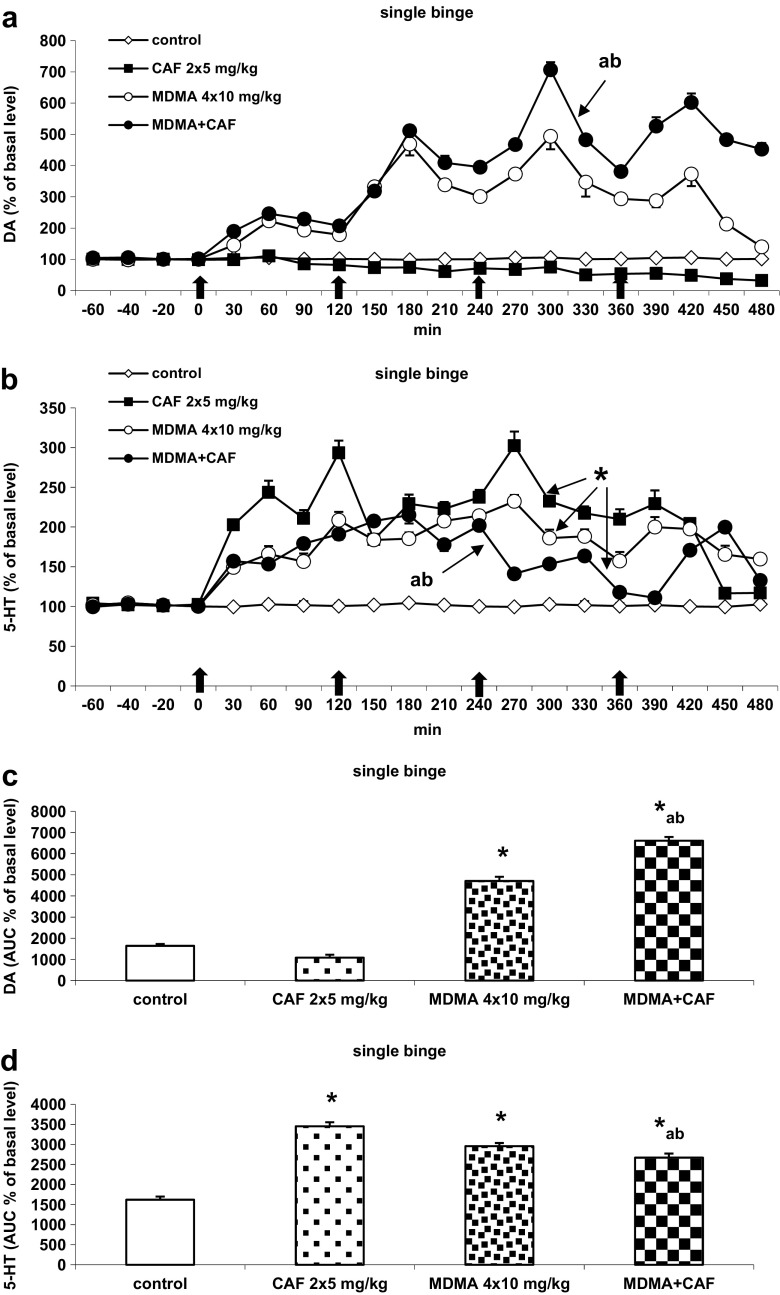
The effect of repeated administration of CAF (2 × 5 mg/kg), MDMA (4 × 10 mg/kg), and their combination on the extracellular level of DA and 5-HT in the mouse striatum. Time of drug injections is indicated with thick arrows. **a**, **b** Time-course. **c**, **d** Total effect expressed as an area under the curve (AUC). Values are presented as the mean ± SEM (*n* = 4 animals per group). Basal extracellular levels of DA and 5-HT were as follows (pg/10 μl): control 1.18 ± 0.06 and 1.67 ± 0.25, CAF 1.42 ± 0.18 and 1.28 ± 0.18, MDMA 2.1 ± 0.23 and 1.27 ± 0.15, and MDMA plus CAF 1.92 ± 0.19 and 1.08 ± 0.09, respectively. *P < 0.0002 vs. control group; “a,” < 0.0002 difference vs. MDMA; “b,” < 0.002 difference vs. CAF (**a**, **b** Repeated-measure ANOVA. **c**, **d** One-way ANOVA and Tukey’s post hoc test)

Caffeine at doses of 5 mg/kg given twice daily 2 hours apart did not affect significantly the basal extracellular DA level (Fig. 1a). MDMA (10 mg/kg) given four times daily 2 hours apart significantly increased extracellular DA level with a maximum after the third injection (Fig. 1a). Co-administration of both drugs produced a significantly stronger effect on extracellular DA level than each of the drugs given separately (Fig. 1a). Repeated-measure ANOVA showed a significant effect of treatment groups [*F*_3,12_ = 158, *P* < 0.0001], sampling period [*F*_15,180_ = 48, *P* < 0.0001], and the interaction between treatment groups and sampling period [*F*_45,180_ = 25, *P* < 0.0001]. The caffeine-induced enhancement of MDMA action on DA release is presented by the total effect expressed as an area under the curve (AUC) in Fig. 1c.

Caffeine given twice daily every 2 hours at a dose of 5 mg/kg markedly increased extracellular 5-HT level in the mouse striatum (Fig. 1b). MDMA (10 mg/kg) administered four times a day every 2 hours increased extracellular 5-HT level to a similar extent as caffeine. However, the increase produced by a combination of both drugs was weaker as compared to the effect of MDMA or caffeine given separately (Fig. 1b). Repeated-measure ANOVA showed a significant effect of treatment groups [*F*_3,12_ = 354, *P* < 0.0001], sampling period [*F*_15,180_ = 25, *P* < 0.0001], and the interaction between treatment groups and sampling period [*F*_45,180_ = 16, *P* < 0.0001]. The MDMA-induced decrease in the caffeine effect on 5-HT release is shown as an AUC in Fig. 1d.

### The Effect of Caffeine on MDMA-Induced Increase in the Extracellular Level of DA and 5-HT in the Mouse Striatum After Acute and Chronic Drug Administrations

**Fig. 2:**
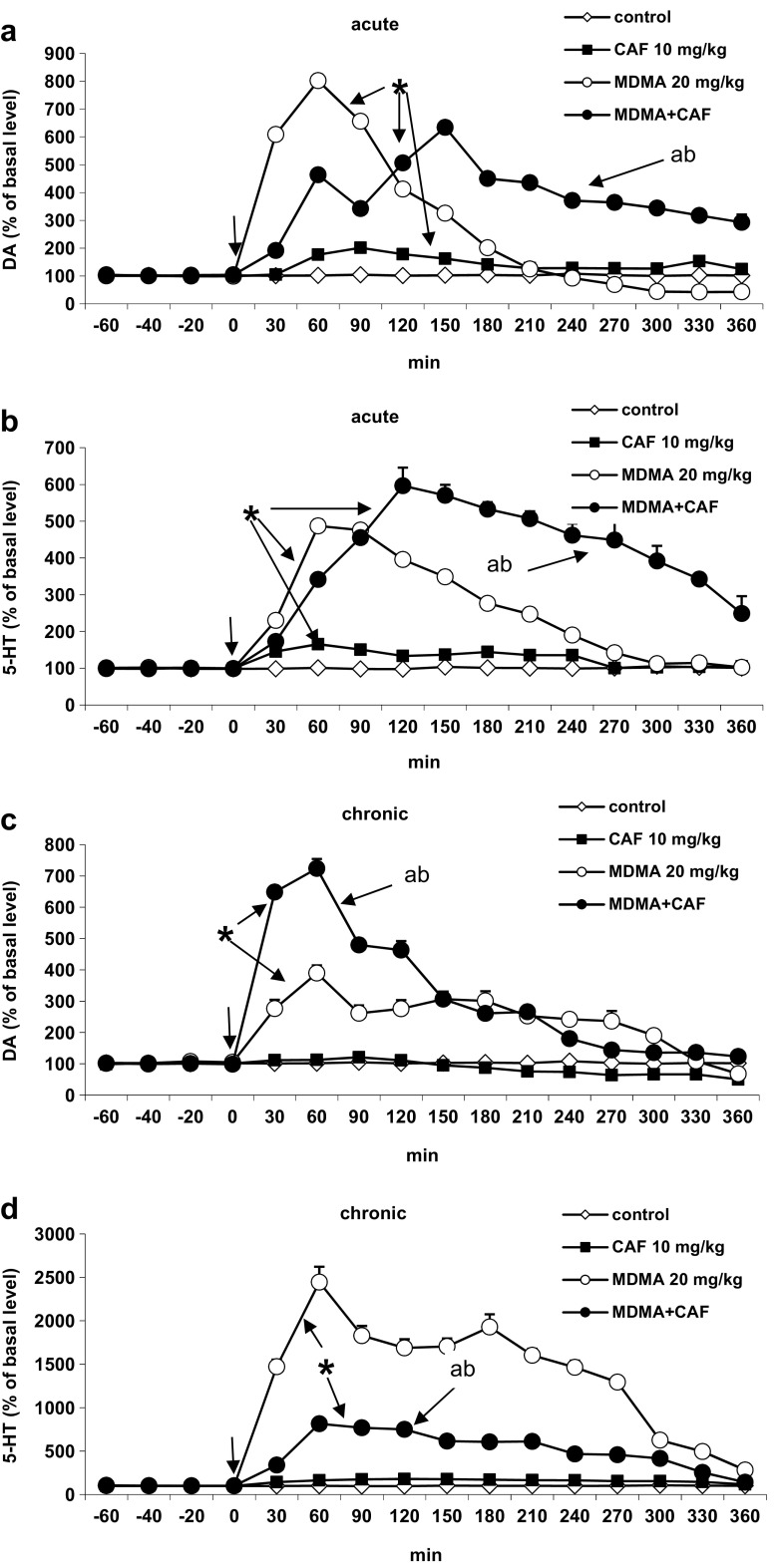
The effect of CAF (10 mg/kg), MDMA (20 mg/kg), and their combination on the extracellular level of DA and 5-HT in the mouse striatum in animals treated chronically with saline (acute) or CAF, MDMA, and MDMA plus CAF (chronic). **a**–**d** Time-course. Values are presented as the mean ± SEM (*n* = 4–8 animals per group). *P < 0.0002 represents a significant difference in comparison to the control group; “a,” < 0.002 difference between MDMA and MDMA plus CAF; “b,” < 0.0002 difference between CAF and MDMA plus CAF (repeated-measure ANOVA and Tukey’s post hoc test)

MDMA at an acute dose of 20 mg/kg increased DA release to ca. 800% of the basal level at 60 min, but the DA release returned to the control values beginning from 210 min after administration (*P* < 0.0002). Caffeine at an acute dose of 10 mg/kg slightly, but significantly, increased DA release and when injected jointly with MDMA shifted the maximum of DA increase to 150 min and prolonged the MDMA-induced increase in DA level until the end of fraction collection time, i.e., 360 min (Fig. 2a). Repeated-measure ANOVA showed a significant effect of treatment groups [*F*_3,14_ = 1311, *P* < 0.0001], sampling period [*F*_11,154_ = 232, *P* < 0.0001], and the interaction between treatment groups and sampling period [*F*_33, 54_ = 215, *P* < 0.0001].

The increase in 5-HT release induced by MDMA at an acute dose of 20 mg/kg was maximal at 60 min after injection, and 5-HT level gradually decreased reaching control values at 300 min of fraction collection. Caffeine at an acute dose of 10 mg/kg slightly but significantly increased 5-HT release and when given jointly with MDMA shifted the maximum MDMA effect to 120 min and prolonged the increase in 5-HT release induced by MDMA until 360 min of fraction collection (Fig. 2b). Repeated-measure ANOVA showed a significant effect of treatment groups [*F*_3,16_ = 260, *P* < 0.0001], sampling period [*F*_11,176_ = 66, *P* < 0.0001], and the interaction between treatment groups and sampling period [*F*_33,176_ = 41, *P* < 0.0001].

The maximal increase in DA release in response to the challenging MDMA dose of 20 mg/kg in animals treated chronically with the drug was lower than that produced by a single dose and amounted to ca. 400% of the basal level (Fig. 2c). Caffeine given chronically did not affect DA release, while it enhanced MDMA effect to ca. 700% of the basal level at 60 min after injection of the challenging dose of 10 mg/kg (Fig. 2c). Repeated-measure ANOVA showed a significant effect of treatment groups [*F*_3,14_ = 178, *P* < 0.0001], sampling period [*F*_11,154_ = 96, *P* < 0.0001], and the interaction between treatment groups and sampling period [*F*_33,154_ = 44, *P* < 0.0001].

The challenging dose of MDMA (20 mg/kg) potently increased 5-HT release in animals treated chronically with MDMA (*P* < 0.0002). The increase in 5-HT release to ca. 2500% of the basal level was maximal at 60 min after injection of the drug (Fig. 2d). The challenging dose of caffeine (10 mg/kg) did not affect 5-HT release in animals treated chronically with this drug, but caffeine inhibited the effect of the challenging dose of MDMA (20 mg/kg) on 5-HT release in animals treated chronically with MDMA plus caffeine (Fig. 2d). Repeated-measure ANOVA showed a significant effect of treatment groups [*F*_3,20_ = 184, *P* < 0.0001], sampling period [*F*_11,220_ = 66, *P* < 0.0001], and the interaction between treatment groups and sampling period [*F*_33,220_ = 40, *P* < 0.0001].

The basal extracellular DA level in the striatum of animals receiving repeated doses of caffeine was significantly (*P* < 0.0001) increased from 2.49 ± 0.21 pg/10 μl (in control) to 6.98 ± 1.21 pg/10 μl of dialysate fraction. In animals receiving repeated doses of MDMA, the basal extracellular level of DA was 4.52 ± 0.87 (*P* < 0.0001 vs. control), while in the group treated chronically with MDMA and caffeine, the basal extracellular DA level was 5.24 ± 0.15 (*P* < 0.0001 vs. control) and was not significantly different from the level in groups receiving the drugs separately.

The basal extracellular level of 5-HT was decreased from 1.25 pg/10 μl in the control group to 0.18 pg/10 μl in the striatum of animals receiving repeated doses of caffeine (*P* < 0.0001). Similarly, chronic administration of MDMA elicited a decrease in 5-HT basal level to 0.19 pg/10 μl (*P* < 0.0001). 5-HT basal level in the group receiving chronically the combination of both drugs was decreased to 0.54 ± 0.05 pg/10 μl (*P* < 0.0001).

Table 1 shows the comparison of the total effect expressed as an AUC in DA and 5-HT release induced by acute and chronic administrations of MDMA and caffeine. It is clear that the effect of the challenging doses of both psychostimulant drugs on DA release was weaker in groups pretreated chronically with caffeine and MDMA vs. their acute doses. In contrast, the effect of the challenging doses of caffeine and MDMA on 5-HT release was stronger in animals receiving chronic treatment of psychostimulants vs. their single doses.

**Table 1:** A comparison of the response to a challenging dose of drugs in animals treated repeatedly with saline (control) or chronically with CAF, MDMA, and MDMA plus CAF with respect to the extracellular levels of DA and 5-HT in the mouse striatum

Treatment (mg/kg)	DA (AUC % of basal level, mean ± SEM)		5-HT (AUC % of basal level, mean ± SEM)	
	Control	Chronic	Control	Chronic
Saline	1232 ± 15	1130 ± 28	1208 ± 31	1047 ± 38
CAF 10	1752 ± 33	1034 ± 23*	1558 ± 22	1908 ± 18*
MDMA 20	3421 ± 52	2909 ± 148*	3121 ± 61	16,831 ± 820*
MDMA 20 + CAF 10	4712 ± 33	3868 ± 78*	5069 ± 217	6239 ± 191*

Values are the mean ± SEM and express an area under the curve (AUC) of the percent of basal level

*P < 0.001 control vs. chronic administration (one-way ANOVA and Tukey’s post hoc test)

### The Effect of Chronic Treatment with Caffeine and MDMA on Tissue Content of DA, DOPAC, HVA, 5-HT, and 5-HIAA in the Mouse Striatum and Frontal Cortex

Chronic treatment with caffeine and MDMA significantly (*P* < 0.001) increased DA content in the mouse striatum, but not in the frontal cortex (Table 2). The increase in DA tissue content was higher in the group treated concomitantly with caffeine and MDMA than in animals receiving these drugs separately (Table 2). Striatal DOPAC tissue level was decreased by caffeine and MDMA, but their combination significantly (*P* < 0.001) reversed this decrease (Table 2). MDMA decreased also DOPAC content in the mouse frontal cortex (*P* < 0.001), but in contrast to the striatum, the decrease in DOPAC tissue level was potentiated (*P* < 0.001) by the combination of caffeine and MDMA (Table 2). 5-HT content was not changed by both psychostimulants and their combination either in the striatum or in the frontal cortex (Table 2). MDMA significantly (*P* < 0.001) decreased the tissue level of 5-HIAA in the striatum and frontal cortex. The decrease in the serotonin metabolite was further potentiated (*P* < 0.001) by the combination of caffeine and MDMA in the striatum, but not in the frontal cortex (Table 2).

**Table 2:** Tissue content of DA, DOPAC, HVA, 5-HT, and 5-HIAA in the mouse striatum and frontal cortex measured 3 h after cessation of treatment with drugs

Treatment	DA	DOPAC	HVA	5-HT	5-HIAA
Striatum (pg/mg wt) ± SEM (*n*)					
Control	9206 ± 568 (11)	913 ± 40 (11)	1016 ± 31 (11)	458 ± 30 (11)	353 ± 16 (11)
CAF	10,929 ± 728 (11)*	631 ± 44 (11)*	1141 ± 95 (11)	495 ± 33 (11)	362 ± 19 (11)
MDMA	11,175 ± 740 (11)*	447 ± 28(11)*	962 ± 35 (11)	435 ± 26 (11)	238 ± 11 (11)*
CAF+MDMA	16,292 ± 2246 (5)*^,^**^,^***	595 ± 74 (5)*^,^**	1169 ± 142 (5)	447 ± 55 (5)	191 ± 18 (5)*^,^**^,^***
Frontal cortex (pg/mg wt) ± SEM (*n*)					
Conrol	1086 ± 90 (11)	133 ± 10 (11)	221 ± 13(11)	450 ± 24(11)	154 ± 7(4)
CAF	1215 ± 121 (11)	130 ± 11 (11)	238 ± 14(11)	435 ± 20(11)	160 ± 10(11)
MDMA	1284 ± 80 (11)	106 ± 8 (11)*	219 ± 15(11)	453 ± 44(11)	129 ± 12(11)*
CAF+MDMA	1224 ± 160 (4)	81 ± 6 (11)*^,^**^,^***	205 ± 14(4)	496 ± 33(4)	130 ± 2(4)*

Values are the mean ± SEM (*n*)

*P < 0.001 vs. control; **P < 0.001 vs. MDMA; ***P < 0.001 vs. CAF (one-way ANOVA and Tukey’s post hoc test)

### The Effect of Chronic Treatment with Caffeine and MDMA on the Density of DAT and SERT in the Mouse Striatum and Frontal Cortex

MDMA given chronically significantly (*P* < 0.001) decreased dopamine and serotonin transporter (DAT and SERT, respectively) densities in the mouse striatum as well as in the frontal cortex (Table 3). The combination of caffeine and MDMA significantly (*P* < 0.001) reversed MDMA-induced decrease in DAT in the mouse striatum while potentiating (*P* < 0.001) the MDMA-induced decrease in SERT in the mouse frontal cortex (Table 3). Caffeine had no influence on DAT and SERT densities in both brain regions (Table 3).

**Table 3:** The density of dopamine transporter (DAT) and serotonin transporter (SERT) in the mouse striatum and frontal cortex measured after cessation of treatment with the drugs

Treatment	DAT	SERT
Striatum density ± SEM (*n*)		
Control	147.7 ± 3.4 (5)	31.7 ± 2.5 (5)
CAF	134.5 ± 4.6 (4)	33.0 ± 0.9 (4)
MDMA	112.4 ± 7.8 (7)*	21.7 ± 1.5 (8)*
CAF+MDMA	137.2 ± 6.9 (7)**	22.1 ± 1.9 (9)*
Frontal cortex density ± SEM (*n*)		
Control	14.0 ± 2.5 (6)	21.2 ± 3.7 (6)
CAF	17.9 ± 3.3 (4)	23.2 ± 0.6 (4)
MDMA	11.1 ± 0.6 (10)*	16.6 ± 2.1 (10)*
CAF+MDMA	11.4 ± 1.1 (8)*	9.5 ± 1.0 (8)*^,^**^,^***

The data are the mean ± SEM (*n*) and express the density of grey values

*P < 0.001 vs. control; **P < 0.001 vs. MDMA; ***P < 0.001 vs. CAF (one-way ANOVA and Tukey’s post hoc test)

### The Effect of Acute and Chronic Treatments with Caffeine and MDMA on the Oxidative Damage of DNA in the Mouse Cortex

**Fig. 3:**
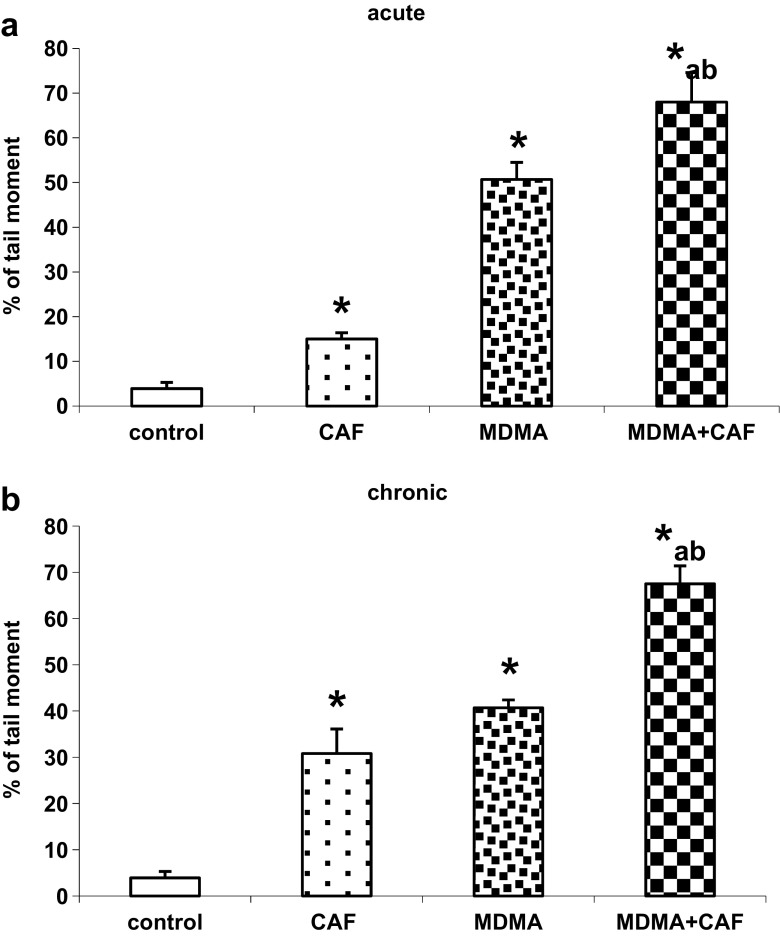
The effect of acute treatment of animals with CAF (10 mg/kg), MDMA (20 mg/kg), and their combination (**a**) or chronic administration of CAF, MDMA, and MDMA plus CAF (**b**) on the oxidative damage of DNA in nuclei from the mouse cortex 2 months after cessation of treatment. Values are presented as the mean ± SEM (*n* = 4–8 animals per group). Data represent an olive tail moment. Olive tail moment is defined as the product of the tail length and the fraction of total DNA in the tail. Tail moment incorporates a measure of both the smallest detectable size of migrating DNA (reflected in the comet tail length) and the number of damaged pieces (represented by the intensity of DNA in the tail). The loss of DNA integrity persisted 60 days after drug administration. *P < 0.01 in comparison to control, “a,” “b” < 0.01 in comparison to MDMA or CAF groups, respectively (one-way ANOVA and Tukey’s post hoc test)

Caffeine and MDMA given acutely or chronically produced oxidative damage of DNA in nuclei from the mouse cortex as measured 2 months after cessation of treatment (Fig. 3a, b). The damage of DNA was stronger after combination of both drugs administered acutely or chronically (Fig. 3a, b). The extent of DNA damage was smaller after all treatments when it was measured 24 h after termination of drug administration (data not shown).

### PDYN and PENK Gene Expressions in the Mouse Striatum After Chronic Treatment with Caffeine and MDMA

**Fig. 4:**
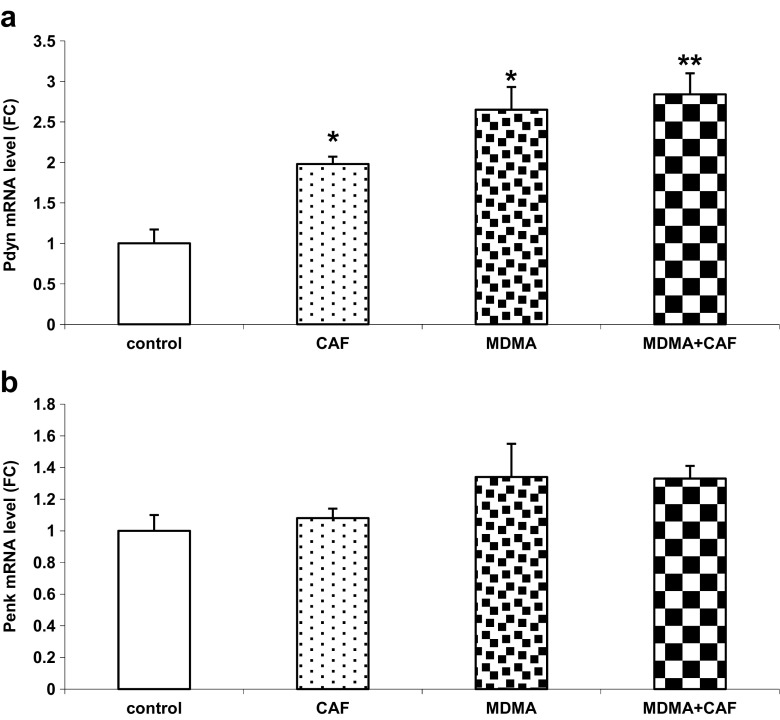
Quantitative real-time PCR analysis of PDYN (**a**) and PENK (**b**) gene expressions in the mouse striatum after chronic administration of CAF, MDMA, and MDMA plus CAF. Values are presented as the mean ± SEM (*n* = 4–10 animals per group). Data represent mRNA levels with respect to the control group. *P < 0.05; **P < 0.001 in comparison to control (one-way ANOVA and Tukey’s post hoc test)

The *Pdyn* mRNA level was significantly increased (*P* < 0.0001) while the *Penk* mRNA remained unchanged in the mouse striatum after chronic administration of MDMA (Fig. 4a, b). Caffeine alone significantly (*P* < 0.05) increased the *Pdyn* but not *Penk* mRNA levels (Fig. 4a, b). The effect of caffeine and MDMA combination on PDYN gene expression did not differ from the effect of MDMA.

### The Effect of Chronic Treatment with Caffeine and MDMA on Exploratory and Locomotor Activities in the Open-Field and Novel Object Recognition Test

**Fig. 5:**
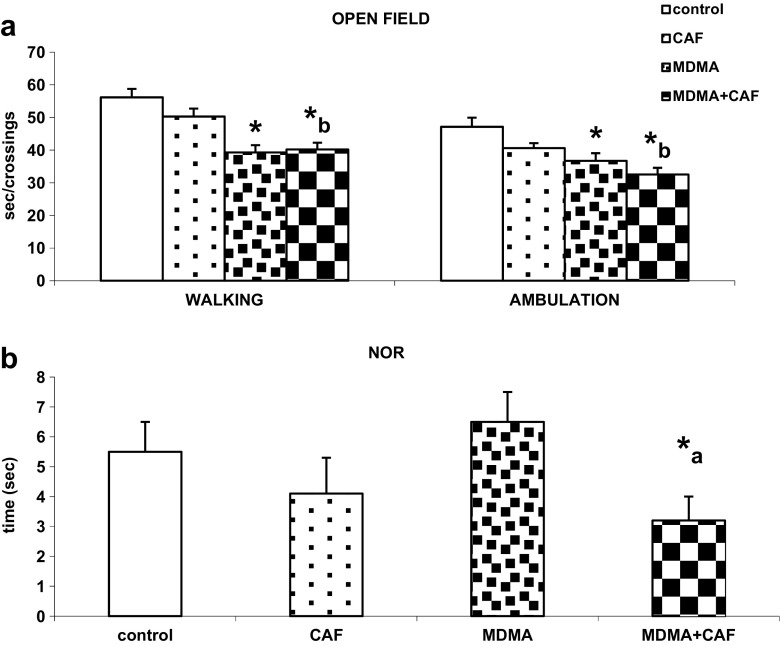
The effect of chronic administration of CAF, MDMA, and MDMA plus CAF on exploratory and locomotor activities in the open-field (**a**) and novel object recognition (**b**) tests. Values are presented as the mean ± SEM (*n* = 10–20 animals per group). Bars represent the time of walking and number of crossings (**a**), and time of exploration of novel object (**b**). *P < 0.01 in comparison to control; “a,” “b” < 0.01 in comparison to MDMA or CAF groups, respectively (one way ANOVA and Tukey’s post hoc test)

MDMA given chronically significantly (*P* < 0.01) decreased the locomotor activity of mice as shown by the decreased time of walking and number of sector crossings in the open field test (Fig. 5a). Caffeine did not affect those parameters. On the other hand, MDMA did not change the time spent by mice on an exploration of the novel object in the novel object recognition test, but when given with caffeine, it significantly decreased this time as compared with control and MDMA-alone groups (*P* < 0.01, Fig. 5b).

## Discussion

Here, we provide an insight into the synergistic interaction between MDMA and caffeine after the “weekend” mode of drug administration in mice. Our findings indicate that caffeine increased the response of DA neurons to the challenging dose of MDMA while decreasing the response of serotonergic neurons. Caffeine potentiated the oxidative damage of nuclear DNA induced by MDMA and had no effect on MDMA-induced decrease in DAT density in the frontal cortex; however, it reversed MDMA-induced DAT decrease in the striatum. Furthermore, caffeine potentiated the decrease in SERT density produced by MDMA in the frontal cortex. Striatal PDYN expression increased by MDMA was not affected by caffeine. Furthermore, exploratory and locomotor activities of mice decreased by MDMA were not affected by caffeine, but exploration of novel object in the NOR test was diminished in animals treated with MDMA and caffeine.

### MDMA Effect on DA and 5-HT Release

In the present study, we report that MDMA produced long-lasting changes in striatal DA and 5-HT release in mice. MDMA used in four repeated sub-maximal doses of 10 mg/kg increased DA and 5-HT release in the mouse striatum to a similar extent (e.g., 400–450% of the basal value), but the effect of the last dose was weaker, and DA level reached the basal value at the end of the experiment. The pattern of 5-HT release was different showing more stable increase throughout the whole collection time. The total effect of MDMA on DA and 5-HT release presented as AUC was comparable. Thus, our in vivo data do not reflect the potency order of DAT and SERT inhibitions by MDMA in rat synaptosomes, where MDMA inhibited SERT stronger than DAT (Rothman et al. 2001). Moreover, MDMA applied in a single higher dose (i.e., 20 mg/kg) was more potent in increasing DA than 5-HT release.

When MDMA was applied chronically (2 days of binge administration per week; this cycle was repeated three times), a weaker response of DA neurons to the challenging dose of MDMA but a stronger response of 5-HT ones was observed. Interestingly, the basal extracellular level of DA in mice receiving MDMA chronically was nearly twofold higher than in control animals, while extracellular 5-HT level was potently decreased. The total effect after chronic MDMA treatment (shown in Table 1) on DA release was reduced by 15% in comparison with control animals, while 5-HT release was potently increased to ca. 540% of control values. It may be speculated that persistent outflow of DA due to loss of DA uptake capacity may be a cause of increased basal extracellular DA level. Considerable evidence from the literature indicates that internalization of DAT occurs in response to amphetamine treatment (Saunders et al. 2000). Thus, decreased surface density of functional DAT may possibly be responsible for increased extracellular DA basal level and weaker effect of MDMA challenging dose on DA release through DAT. In fact, we observed a decrease in DAT density in the striatum and frontal cortex following chronic exposure to MDMA, which is in line with studies of Saunders et al. (2000). In contrast to the effects of MDMA on DA release, the basal extracellular 5-HT level was strongly suppressed in animals treated chronically with MDMA, but response to the challenging MDMA dose was very potent. This is probably due to compensatory upregulation of 5-HT release machinery resulting from low synaptic 5-HT levels. It is not clear whether a low basal extracellular 5-HT level in animals treated chronically with MDMA results from dysfunctional SERT. In the study with MDMA self-administration (Schenk et al. 2007) or its repeated application (Xie et al. 2006), a decrease in SERT was noted. Our data also show MDMA-induced decrease in SERT density in the striatum and frontal cortex of mice after its chronic administration. Since the loss of surface expression of SERT correlates with lowered 5-HT basal level, it may be speculated that 5-HT neuron terminals may be damaged by MDMA.

The different effect exerted by MDMA on 5-HT and DA has been revealed in a number of studies. For instance, Koch and Galloway (1997), Reveron et al. (2010), and Shankaran et al. (1999) found that depending on the dose, MDMA was several times more potent in influencing synaptic 5-HT than DA. The effect of MDMA on DA and 5-HT systems may be due to modulation of different receptors. Activation of 5-HT1A and 5-HT2A/2C receptors localized on DA cells and neuronal terminals or on GABA or glutamate neurons by 5-HT or directly by MDMA (Di Mateo et al. 2008) exerted rather inhibitory control on DA. However, some studies report that the activation of 5-HT1A (Ichikawa et al. 2001; Rollema et al. 2000), 5-HT1B (Yan and Yan 2001; O’Dell and Parsons 2004), or 5-HT2A receptors in the frontal cortex (Pehek et al. 2006) increased DA release, whereas activation of 5-HT2C receptors was inhibitory to DA (Bankson and Yamamoto 2004). Thus, it may be suggested that long-term exposure to MDMA leads to neuroadaptative changes in sensitivity of serotonin receptors, which may result in differential response of DA and 5-HT neurons, as it is observed in our study. On the other hand, MDMA acting by itself or via SERT-released 5-HT may affect other neurotransmitter systems. Some data suggest a role of postsynaptic 5-HT2A receptors located on glutamatergic neurons in the neurochemical effects mediated by MDMA. Stimulation of 5-HT2A receptors located on glutamatergic cells in the frontal cortex may elicit an increase in glutamate level leading indirectly to a rise in DA and 5-HT release (Alex and Pehek 2007). MDMA may also suppress nigral GABA release following 5-HT2A/2C receptor activation thus causing disinhibition of the striatal DA neurons (Gudelsky and Yamamoto 2008). One can speculate that the observed changes in 5-HT2A/C receptors may represent neuroadaptative responses secondary to changes in SERT expression induced by MDMA. However, it remains unclear how glutamate and GABA release may be involved in upregulation of serotonergic neurons and cause very potent response to the challenging dose of MDMA, as we observed in the present study.

### MDMA and Caffeine Co-administration Effect on DA and 5-HT Release

Caffeine given repeatedly increased the basal extracellular level of DA and increased MDMA effect on DA release. However, in contrast to animals pretreated with saline in which caffeine potentiated MDMA-induced increase in 5-HT release, caffeine inhibited the MDMA effect on 5-HT release in animals receiving both psychostimulants repeatedly. It is accepted that the mechanism underlying caffeine influence on neurotransmitter release is related to the blockade of adenosine A1 and A2A receptors. Caffeine may increase DA and glutamate release in the striatum via blockade of inhibitory A1 receptors as was evidenced by a number of studies (Borycz et al. 2007; Ciruela et al. 2006; Górska and Gołembiowska 2015; Okada et al. 1996). Similarly, adenosine A1 receptor blockade was shown to increase striatal and hippocampal 5-HT release (Górska and Gołembiowska 2015; Okada et al. 1999). There is also considerable evidence that A2A receptors highly expressed in striatopallidal GABAergic neurons (Ferré et al. 1993) and on striatal glutamatergic terminals (Ciruela et al. 2006) are involved in the enhancement of extracellular DA and 5-HT levels by caffeine. The lack of A2A receptors on the striatal monoaminergic neuronal terminals suggests that their role in the control of DA and 5-HT release may be secondary and related to the changes in the activity of striatal output pathways elicited by postsynaptic A2A receptors. In support of this concept, there are studies showing that the administration of A2A adenosine receptor antagonists increased DA release and 5-HT release in the striatum of rats and mice (Gołembiowska et al. 2009, Górska and Gołembiowska 2015; Okada et al. 1996).

Chronic co-administration of caffeine and MDMA produced a stronger effect on DA release as compared to MDMA alone in response to the challenging doses of both psychostimulants. These data indicate synergistic interaction between caffeine and MDMA. It may be postulated that caffeine by blocking A1 and A2A receptors and MDMA via DAT could increase DA release in chronically pretreated animals. Our earlier study with a single-dose drug treatment indicates that caffeine-potentiated MDMA evoked DA release by blockade of A1 and A2A receptors (Górska and Gołembiowska 2015). However, other authors reported that the exacerbation of MDMA effect by caffeine in striatal slices was mediated via A1 receptors blockade (Vanattou-Saïfoudine et al. 2011). The difference in results reported by the above-cited studies may be related by way of drug application (systemic vs. local) and animal species (mice vs. rats).

The diminished response of 5-HT neurons after co-administration of caffeine and MDMA as compared to MDMA alone is difficult to explain. It may be speculated that persistent blockade of adenosine receptors by caffeine can be responsible for this effect. It was demonstrated that adenosine A1, A2, and A3 receptors could modulate hippocampal 5-HT release (Okada et al. 1999). In the study of Okada et al. (1999), the stimulatory effects of A2 and inhibitory effects of A3 receptors on 5-HT release were abolished by A1 receptor activation. However, under A1 receptor blockade by caffeine, the inhibitory effects of A3 receptor were unmasked in addition to the effect of A2 receptor blockade by caffeine (Okada et al. 1999, 2001). Furthermore, it was also demonstrated that 5-HT reuptake activity might be modulated by A3 receptor (Okada et al. 2001). Thus, the described mechanism of adenosine receptor involvement in the control of 5-HT release and their blockade by caffeine may be responsible for the diminished 5-HT release in response to MDMA and caffeine co-administration.

### Neurotoxic Effects of MDMA and Caffeine

The neurotoxic effect of MDMA in mice seems to be related to dopaminergic and serotonergic systems. Our data showed a significant decrease in markers of neuronal terminals, DAT, and SERT in the mouse striatum and frontal cortex after chronic administration of MDMA. There was also depletion of tissue concentration of DOPAC and 5-HIAA, but not DA and 5-HT, in the striatum and the frontal cortex of mice. Neurotoxic effect seems to result from formation of ROS because we observed oxidative damage of neuronal DNA in the cortex 2 months after acute and chronic doses of MDMA. In addition, the formation of free radicals after MDMA administration in the mouse striatum was evidenced in our earlier study (Górska et al. 2014). The role of ROS in MDMA neurotoxicity was also demonstrated in many other works. Free radical formation by MDMA was found in the mouse striatum by Colado et al. (2001). The involvement of free radicals in MDMA-induced dopaminergic neurotoxicity in mice was also shown by Peraile et al. (2013). Those authors demonstrated that oxidative stress was related with lipid peroxidation and with an increase in superoxide dismutase and decrease in catalase activity. Hydroxyl radical formation together with products of tryptamine oxidation was proposed as the mechanism of MDMA-induced depletion of brain 5-HT by Shankaran et al. (1999). Moreover, it was suggested that 5-HT depletion was dependent on 5-HT transporter activity. It is speculated that DA released by MDMA may enter the 5-HT terminal through SERT and may be oxidized by MAO-B, which is present in 5-HT terminals, leading to the generation of free radicals in 5-HT neurons (Falk et al. 2002; Shankaran et al. 1999). Barbosa et al. (2012) indicated that MDMA metabolites, in particular α-methyl-dopamine, together with high levels of monoamine neurotransmitters might be the major contributors to MDMA neurotoxic effects.

Oxidative damage produced by MDMA may be associated with neuronal cell bodies. As shown in our work, MDMA caused single- and double-strand breaks in cortical nuclear DNA. Reactive oxygen species, in particular hydrogen radical, formed even after a single dose of MDMA, are able to interact with DNA bases. In our study, oxidative stress triggered DNA alterations which persisted for 2 months after single or repeated doses of MDMA. Most of the literature has described the striatum as the main target of MDMA neurotoxicity. Here, we provide evidence that other regions are also targets of MDMA neurotoxicity. DNA damage may be a molecular basis for MDMA-induced neuroplasticity with subsequent behavioral and cognitive deficits. In our study, caffeine co-administered with MDMA promoted damage of nuclear DNA 2 months after termination of the treatment; thus, it enhanced oxidative stress induced by MDMA. Caffeine also enhanced the decrease in cortical DOPAC and 5-HIAA in the striatum and frontal cortex. At the same time, it slightly but significantly reversed the decrease in striatal DOPAC content and increased DA striatal tissue level. It can be concluded that caffeine increased MDMA-induced deficit in striatal 5-HT neurons and DA deficit in the frontal cortex. However, it was neuroprotective for DA fibers in the striatum. These data partially correspond to our results with measurement of SERT and DAT fiber densities. In these experiments, caffeine reversed the decrease in striatal DAT-positive fibers in animals treated with MDMA. However, it had no effect on DAT- and SERT-positive fibers in the frontal cortex and striatum, respectively. On the other hand, it potentiated the decrease in cortical SERT fiber density. It has to be pointed out that reversal of MDMA-induced decrease in DAT fiber density by caffeine corresponds to the caffeine-induced enhancement of the effect of MDMA on DA release in the striatum. Thus, caffeine seems to be neuroprotective for striatal dopaminergic fibers, but it seems to increase neurotoxic damage of cortical 5-HT terminals. Moreover, besides damage of cortical 5-HT terminals, caffeine increased MDMA-induced oxidative damage of cortical DNA which suggests degeneration of neuronal cells in this brain region. The neurotoxic effect exerted on cortical neuronal cell bodies may lead to neuroadaptive change of cortical pathways projecting to nigral or raphe nuclei. Thus, overall caffeine effect seems to be partly neuroprotective and partly neurotoxic. This dual action is dependent on doses, the brain region examined, and the schedule of administration as reported by numerous literature. Caffeine has been shown to have neuroprotective properties in the animal model of Parkinson’s disease (PD) (Kalda et al. 2006) and decreased the risk of development of PD as was demonstrated by several epidemiological studies (Ascherio et al. 2001; Ross et al. 2000). When caffeine was given chronically, it increased activity of antioxidant enzymes superoxide dismutase (SOD) and catalase (CAT) in several regions of the rat brain (Noschang et al. 2009a). Aoyama et al. (2011) evidenced that caffeine induced neuronal glutathione (GSH) synthesis by promoting cysteine uptake in mouse hippocampal pyramidal neurons. Chronic caffeine ingestion reduced the lipid peroxidation and increased GSH level and SOD activity in the rat brain (Abreu et al. 2011). On the other hand, caffeine exhibited prooxidant properties in vitro (Azam et al. 2003) and inhibited DNA repair mechanisms (Selby and Sancar 1990). The neurotoxic potential of caffeine given acutely was evidenced in the mouse brain by enhanced astroglia and microglia reactivities by MDMA (Khairnar et al. 2010). In contrast, chronic low doses of caffeine exerted anti-inflammatory effects and prevented MDMA-induced neuroinflammatory reaction (Ruiz-Medina et al. 2013). Study of Frau et al. (2016) showed that caffeine potentiated MDMA-induced DA neuron degeneration and neuroinflammation in adolescent mice. Vanattou-Saïfoudine et al. (2012) proposed that caffeine exacerbated adverse reactions induced by various psychostimulants, such as hyperthermia, tachycardia, and increased mortality as the result of increased DA release. Thus, caffeine shows differential effects, neuroprotective or neurotoxic, when co-administered with MDMA indicating that the mechanism of action of psychoactive drug combination needs further clarification.

### MDMA and Caffeine Effects on PDYN and PENK Gene Expressions and on Behavior

In our study, chronic MDMA increased PDYN gene expression in medium spiny GABA neurons expressing D1 receptor, while it did not affect PENK expression in GABA neurons projecting to the globus pallidus expressing D2 receptor. This is in line with findings of Benedetto di et al. (2006), who reported an increase in PDYN expression in the rat striatum after chronic administration of MDMA. These data confirm also the role of D1 receptor in MDMA effects, in particular in the development of neurotoxicity after long-term administration (Granado et al. 2014). The overstimulation of a direct GABAergic pathway with normal functioning of an indirect GABAergic pathway may be responsible for deficit in locomotor activity of mice observed in our study. The lack of caffeine influence on MDMA-induced PDYN expression seems to correlate with its lack on MDMA-induced locomotor activity of mice in the open-field test. As mentioned in the “Introduction” section, caffeine increases the activity of both types of neurons (Johansson et al. 1994). Thus, balanced effect on both GABAergic pathways by caffeine may underlie the lack of influence on the PENK expression. On the other hand, caffeine co-administered chronically with MDMA decreased the time of exploration of unknown object in the novel object recognition test. It is likely that a combination of both psychostimulants induced deficit in cognitive functions of mice, as also demonstrated by Costa et al. (2014). Structural synaptic plasticity of the medial prefrontal cortex was correlated with changes in response to novelty in rats developmentally treated with cocaine (Caffino et al. 2017). It may be suggested that damage of serotonergic terminals in cortical regions or possible oxidative damage of glutamatergic pathways projecting to the VTA or raphe cell bodies may be responsible for the effect of psychostimulants on cognitive functions. The role of glutamatergic pathway damage by oxidative stress in the hippocampus and cognitive impairment was also shown in mice by Frenzilli et al. (2007). Anxiety-like behavior in rats was related to oxidative damage of DNA in the hippocampus by chronic caffeine (Noschang et al. 2009b). The alterations in the brain antioxidant system were suggested to affect the cognitive functions of rats after chronic caffeine ingestion (Abreu et al. 2011).

### Conclusions

In conclusion, our data provide evidence that long-term caffeine administration has a powerful influence on dopaminergic and serotonergic neuron functions disturbed by MDMA in the mouse brain and on neurotoxic effects evoked by MDMA. Caffeine potentiates MDMA effect on dopaminergic system and inhibits its effect on serotonergic neurons. Exacerbation of MDMA-evoked oxidative stress may cause damage of serotonergic terminals.
